# Geminivirus Suppressor Proteins: Targeting Host DNA Methylation Machinery

**DOI:** 10.3390/biology15212030

**Published:** 2026-11-07

**Authors:** Minenhle Felicia Zenda, Farhahna Allie, Abidemi Paul Kappo

**Affiliations:** Department of Biochemistry, Faculty of Science, University of Johannesburg, Auckland Park, Johannesburg 2006, South Africa; zendaminenhle@gmail.com (M.F.Z.); fallie@uj.ac.za (F.A.)

**Keywords:** geminvirus, RNA-directed DNA methylation, DNA methylation epigenetics, RdDM suppression, replication-associated protein, transcription activator protein

## Abstract

Geminiviruses are a large family of DNA viruses that are a major threat to agricultural biosecurity. They are obligate pathogens that replicate using Rolling circle replication (RCR). During RCR, their double-stranded DNA intermediates become targets of host-mediated RNA-dependent DNA methylation (RdDM), an important epigenetic antiviral defense mechanism. However, geminiviruses encode multifunctional proteins that suppress and counteract transcriptional gene silencing. This review examines four geminiviral multifunctional proteins, the replication-associated protein (C1), the transcriptional activator protein (C2), the movement protein (V2), and the C4 protein, that suppress DNA methylation. These viral proteins interfere with DNA methylation through several mechanisms, including disrupting key components such as Argonaute 4 (AGO4) and Domain Rearranged Methylase 2 (DRM2); repressing methylation-associated genes transcriptionally; and indirectly hindering SAM production by disrupting the methyl cycle. Despite these advances, the mechanism by which DNA methylation restricts viral gene expression and may limit viral replication remains poorly understood. The interplay between geminviruses and host RdDM exemplifies a molecular arms race, in which RdDM serves as a critical defense actively targeted by geminvirus suppressor proteins.

## Simple Summary

Geminiviruses are a family of DNA viruses that threaten agricultural and economically important plants. Plants have developed various mechanisms to suppress viral infection. DNA methylation is a defense mechanism plants use to block viral gene activity. However, geminiviruses produce proteins that disrupt this defense to establish infection. This review explores how geminivirus proteins interfere with DNA methylation and related cellular processes to overcome plant antiviral defenses.

## 1. Introduction

Plants survive various environmental challenges through a multilayered defense strategy that includes structural, developmental, physiological, and epigenetic adaptations. Unlike structural and physiological adaptations, epigenetic responses are dynamic, potentially reversible, and responsive to environmental stimuli. Epigenetics examines differences in heritable and non-heritable epigenomic states, such as histone modifications and DNA methylation, that occur independently of the underlying DNA sequence and influence gene expression [1]. Epigenetic mechanisms govern much of the plant’s biological function, including development, regulation of the cellular environment, and defense responses. In plants, DNA methylation is one of the most extensively studied epigenetic mechanisms. It regulates gene expression, controls transposons, and maintains genome integrity by adding methyl groups to cytosines in DNA [2]. This epigenetic mark is associated with gene regulation, transposon silencing, genome stability, and genomic imprinting.

DNA methylation occurs in distinct sequence contexts that vary among organisms. The most common type of DNA methylation in complex eukaryotes is 5-methylcytosine (m^5^C), but N4-methylcytosine (m^4^C) and N6-methyladenine (6mA) have been rarely reported. Recently, Walker et al. [3] reported extensive m^4^C methylation at the last stage of spermiogenesis in *Marchantia polymorpha* (liverwort). This rare type of DNA methylation is facilitated by the specific *M. polymorpha* gene DNA N4 cytosine methyltransferase1a (MpDN4MT1a), which phylogenetically clusters with other prokaryotic N4 cytosine methyltransferases and contains the TSPPY sequences characteristic of the N^4^ methyltransferase catalytic domain. Deletion of the MpDN4MT1a gene reduced sperm mRNA abundance, retarded swimming behaviour, and caused abnormal embryo development [1]. With advances in high-throughput technologies, researchers have reported 6mA in plants and other eukaryotes [4]. Unlike animals, where DNA methylation occurs predominantly at the CG dinucleotide, plants methylate CG, CHG, and CHH contexts (where H denotes nucleotides A, C, or T). Targeted mutations in genes involved in DNA methylation often cause developmental abnormalities in model plants, such as *Arabidopsis thaliana*, *Oryza sativa L*., and *Solanum lycopersicum* [5,6,7]. More specifically, plants use *de novo* methylation, known as RNA-directed DNA methylation (RdDM), to inhibit DNA viral replication. For instance, the V1/V2 and C1/C4 overlapping open reading frames of the Tomato curly stunt virus genome are hypermethylated in tolerant plants [8].

RNA-directed DNA methylation (RdDM) is a plant-specific epigenetic mechanism that uses small interfering RNAs (siRNAs) as guides for *de novo* DNA methylation, often resulting in transcriptional gene silencing without altering the genetic sequence. RdDM functions through canonical and non-canonical pathways that differ in their initiation mechanisms. In the canonical pathway, the chromatin reader SAWADEE HOMEODOMAIN HOMOLOG 1 (SHH1) recognizes and binds methylated K9 and unmethylated K4 on the histone H3 tail. SHH1 is a small protein that plays a significant role in the biogenesis of 24-nucleotide (nt) RNAs. The SAWADEE domain of SHH1 adopts a Tudor-like fold that preferentially recognizes H3K9 methylation and unmethylated H3K4 [9]. Once anchored to the RdDM target loci, RNA polymerase (Pol IV) is recruited and transcribes the H3K9-methylated chromatin regions into precursor RNA. RNA-dependent RNA polymerase 2 (RDR2) and Dicer-like 3 (DCL3) process precursor RNA into double-stranded RNA and 24-nt small interfering RNA (siRNA) [10,11]. Next, HUA Enhancer 1 (HEN1) stabilises the 24-nt siRNAs by methylating the 3′-OH. The methylated siRNAs are loaded into Argonaute (AGO) 4 and 6. AGO4/AGO6, together with Pol V-derived scaffold transcripts, recruit DOMAINS REARRANGED METHYLTRANSFERASE 2 (DRM2) to catalyze de novo DNA methylation. Methyltransferase 1 (MET1) and Chromomethylase 3 (CMT3) maintain symmetric methylation at CG and CHG islands, while DRM2 and CMT2 maintain asymmetric methylation. Together, these enzymes maintain DNA methylation across CG, CHG, and CHH sequence contexts. In the non-canonical pathway, primary siRNAs, miRNAs, and siRNAs are synthesized from miRNA precursors, transposable elements, non-coding RNAs, and inverted repeats. Unlike the canonical pathway, 21-nt siRNA generated by other plant machinery, for example, RNA polymerase II transcription, triggers the non-canonical pathway. This pathway ultimately converges with the canonical RdDM pathway through shared DCL and AGO components [12].

## 2. DNA Methylation as a Defense

DNA methylation and its quantification were first reported in the 1900s. Early studies by Wyatt [13] provided some of the first estimates of m^5^C abundance in nucleic acids, although modern technologies have substantially improved detection accuracy. Advancements in omics-based technologies have enabled precise quantification and identification of nucleotide base methylation, including Methylation-Specific PCR (MSP), bisulfite sequencing, and mass spectrometry-based approaches (LC-MS/MS and MALDI-TOF MS). Recently, Sharma et al. [14] identified a potential function of endogenous geminiviral sequences in plants, reporting that a large-scale survey of green plants revealed that 42 angiosperm species harbour geminivirus replication-associated protein (Rep) homologs. Endogenous geminivirus-like (EGV) elements are small geminivirus-derived DNA sequences that RdDM and other heterochromatic mechanisms silence. EGVs have been integrated into the plant’s genome over evolutionary time and are unlikely to be infectious. They have been identified in the genomes of *Dioscorea* spp. (yams), *Malus domestica* (apples), *Lactuca sativa* (lettuce), *Populus trichocarpa* (cottonwood), and *Coffea canephora* (coffee) [15,16,17]. EGVs identified in *Solanum melongena* (eggplant), *Nicotiana tabacum* (tobacco), and *Camellia* spp. have been reported to be transcriptionally active [14,17,18]. DNA methylation studies on EGVs in *Camellia sinensis* var. assamica and *N. tabacum* showed high methylation levels, suggesting transcriptional gene silencing via the RdDM pathway, which may aid in defense against geminiviruses [14]. Methylation-mediated silencing of endogenous geminiviral sequences further supports the idea that host epigenetic pathways can recognize and repress geminivirus-derived DNA.

*Geminiviridae* is a family of DNA viruses that infect economically and agriculturally important crops. The most significant agricultural damage has been reported in tropical and subtropical regions worldwide, with Asia, America, and Africa affected by more genera [19,20]. The virus family comprises more than 500 species that exist as either monopartite or bipartite twinned virions, each encapsulated in an icosahedral capsid [19,21]. Members of the family *Geminiviridae* span 15 genera, as inferred from genomic organization, phylogenetic reconstruction, host range, and insect vectors [22]. Their genomes contain up to 8 open reading frames, but encode only one structural protein [23]. Geminiviruses encode multifunctional proteins that facilitate cell-to-cell movement, replication, symptom determination, and suppression of defense responses. Geminivirus infection activates multiple host defense pathways, including RNA silencing, immune signalling, and epigenetic defenses such as DNA methylation.

### High-Throughput Sequencing Reveals Methylation Patterns in Geminiviral Genomes

Advances in high-throughput sequencing have enabled more detailed characterization of DNA methylation in geminiviruses, providing sequence-level evidence that complements biochemical investigations of RNA-directed DNA methylation (RdDM) and viral suppressor activity. Mulaudzi et al. [8] used bisulfite conversion, PCR amplification and next-generation sequencing to characterize the methylation profile of Tomato curly stunt virus (ToCSV) in tolerant and susceptible tomato lines. Although overall viral DNA methylation was low (<3%), methylation was more pronounced in tolerant plants across CG, CHG and CHH sequence contexts. Differentially methylated cytosines were concentrated primarily in the overlapping V2/V1 and C4/C1 genomic regions, while the 3′ intergenic region exhibited significant differences in CG methylation. These findings demonstrate that methylation patterns vary across the viral genome and between host phenotypes, supporting a role for host-mediated epigenetic regulation in the tomato–ToCSV interaction [8].

Complementary evidence comes from studies combining methylation-dependent restriction with deep sequencing of geminiviral minichromosomes. Differential methylation of circular DNA in geminiviral minichromosomes revealed a non-uniform distribution of methylated sites, including enrichment in particular genomic regions associated with nucleosome organization [24]. These approaches provide higher-resolution methylation patterns that may be relevant to viral chromatin structure and gene regulation.

Similarly, bisulfite sequencing has also been used to investigate the relationship between viral suppressor activity and DNA methylation. Gui et al. [25] demonstrated that the betasatellite-encoded βC1 protein promotes the demethylation of Tomato yellow leaf curl China virus (TYLCCNV) DNA through interactions with host DNA glycosylases. Coinfection with the betasatellite was associated with reduced viral DNA methylation, whereas suppression of host DNA demethylases restored methylation. These findings provide sequence-level evidence linking a defined viral counter-defense mechanism to changes in the viral genome’s methylation state.

Collectively, these studies demonstrate how sequencing-based approaches complement biochemical evidence of geminiviral suppression of host methylation pathways. However, methylation patterns alone do not establish the causal contribution of individual suppressor proteins, and the extent to which methylation influences viral transcription and replication remains context-dependent. Further integration of high-resolution methylation profiling with viral gene-expression measurements, chromatin analyses and functional characterization of suppressor proteins will help clarify the relationship between RdDM suppression and geminivirus infection.

## 3. Geminivirus and DNA Methylation

Geminivirus replication occurs in the plant cell’s nucleus following rolling circle replication (RCR). During geminivirus RCR, double-stranded DNA replicative intermediates (open circular, covalently closed circular (cccDNA), and heterogeneous linear DNA) serve as templates for replication and transcription of the viral genome and proteins. The cccDNA forms viral minichromosomes by associating with cellular histone proteins [26,27]. Begomovirus-derived nucleosomes are covered by 11,12 nucleosomes. Minichromosomes with 12 nucleosomes have an open gap that supports binding of host transcription factors and are transcriptionally active [27]. These minichromosomes carry markers associated with chromatin activation and repression, such as histone H3K9 methylation and DNA methylation [28]. Although it is unclear how geminiviruses trigger RdDM, components of the RdDM pathway have been implicated in establishing histone modifications and generating 24-nt viral siRNAs (vsiRNAs) [29,30]. Evidence that DNA methylation restricts geminivirus infection includes increased susceptibility in plants carrying mutations in RdDM-associated genes, reduced viral replication in protoplasts transfected with pre-methylated viral DNA, elevated m5C levels in plants recovering from geminivirus infection, and enhanced resistance in TY-1 introgressed tomato lines. TY-1 encodes an RNA-dependent RNA polymerase of the RDRγ class that promotes viral siRNA accumulation and enhances methylation of viral genomes, thereby strengthening antiviral defense against several begomoviruses [31,32]. However, eukaryotic viruses such as geminiviruses have evolved suppressor proteins that reduce the effectiveness of host methylation-based defense mechanisms. Although numerous studies demonstrate a negative association between viral DNA methylation and geminivirus accumulation, the relative contributions of transcriptional repression, chromatin compaction, and direct inhibition of replication remain incompletely resolved. Consequently, geminiviruses have evolved multiple suppressor proteins that target distinct components of the host methylation machinery to overcome these antiviral defenses (Table 1).

### 3.1. Replication-Associated Protein (C1)

The complex interplay between host-induced transcriptional gene silencing and active viral counter-defenses regulates viral replication and transcription. Host-derived DNA methylation suppresses viral gene transcription, thereby limiting the accumulation of viral elements and indirectly reducing viral replication [28,33]. The geminivirus replication initiator protein is vital for viral replication and transcription. During replication, the C1 protein is transcribed early from the intergenic region (IR), where the C1 protein’s core promoter and the virus’s origin of replication are located. Thereafter, it initiates replication of the viral genome. Although C1 is primarily required for initiation and maintenance of viral DNA replication, accumulating evidence indicates it also helps suppress host methylation-based antiviral defenses. The protein is typically 320–400 amino acids long and has a molecular mass of about 41 kDa. The Begomovirus C1 protein contains an oligomerization domain with two enzymatic domains: the N-terminal domain, a DNA-binding domain responsible for endonuclease and ligase activities, and the C-terminal domain, a helicase domain with an embedded ATPase Motif [19,23,34]. The N-terminal domain contains 3 motifs: Motifs I, II, and III. A conserved geminivirus sequence (GRS) is also located between motifs II and III [35]. The multifunctional protein forms homo- and hetero-oligomeric complexes with viral and host proteins to facilitate replication and regulate host cellular processes.

During replication, the various synthesized replication forms are methylated according to their conformation, with heteroduplex dsDNA preferentially methylated, whereas other replication forms are largely unmethylated [36]. C1 remodels viral minichromosomes and is associated with the ubiquitination machinery. The C1 protein from the Chilli leaf curl virus (ChiLCV) co-localizes and interacts with UBIQUITIN-CONJUGATING ENZYME2 (NbUBC2; E2) and HISTONE MONOUBIQUITINATION1 (NbHUB1; E3) [37]. The interaction between C1 and NbUBC2 caused NbUBC2 to re-localize to the nucleus. Although C1 interacts with NbUBC2 and NbHUB1, it is not ubiquitinated; instead, this interaction enhances deposition of the active histone mark monoubiquitinated histone H2B (H2B-ub). H2B-ub is deposited on the ChiLCV in the promoter region of the minichromosomes and promotes trimethylation of histone 3 at lysine 4 (H3K4me3), resulting in a transcriptionally active minichromosome. C1-dependent enrichment of H2B-ub and H3K4me3 promotes a transcriptionally permissive chromatin state, generally associated with reduced DNA methylation and enhanced viral gene expression. One possibility is that C1-mediated chromatin remodelling alters viral DNA accessibility to host methylation machinery, although the precise mechanism remains unclear. Rodríguez-Negrete et al. [38] further showed that C1 suppresses MET1 and CMT3 transcription, linking viral infection directly to reduced host methyltransferase expression.

Similarly, Beet severe curly top virus (BSCTV) C1 protein causes an ectopic expression of VARIANT IN METHYLATION5 (VIM5) [39]. VIM5 is a methylcytosine-binding protein that was previously not identified as a key functional member responsible for maintaining global CG methylation, as transcript quantification showed little or no expression in Arabidopsis wild-type plants grown under normal conditions [40]. In BSCTV-infected Arabidopsis plants, VIM5 acts as an E3 ubiquitin ligase and physically associates with and ubiquitinates the methyltransferases MET1 and CMT3. This interaction targets these methyltransferases for degradation via the 26S proteasome, leading to loss of symmetric methylation at the viral DNA promoter and promoting transcription of the early viral genes C2 and C3 [39]. Collectively, these findings suggest that C1 suppresses host methylation-based defenses through multiple complementary mechanisms, including transcriptional repression of methyltransferase genes, chromatin remodelling of viral minichromosomes, and targeted degradation of methylation machinery (Table 2).

### 3.2. C4 Protein

The A/C4 is the smallest protein encoded by geminivirus. It is designated AC4 in bipartite viruses and C4 in monopartite viruses. Its coding region is completely embedded within the larger Rep open reading frame. More specifically, it is overprinted on the Rep ORF, which encodes the DNA-binding and oligomerization domains [23]. Topilevirus, Grablovirus, and Capulavirus encode a C3 protein, a C4 homolog. This protein ranges from 5.3 kDa in Maize streak virus (MSV) (South African Isolate) to 18.1 kDa in Tomato apical leaf curl virus [41]. The AC4/C4 protein lacks a well-defined structure because it is entirely disordered. This flexibility allows the protein to bind various proteins. The A/C4 protein can cause developmental abnormalities, such as hyperplasia, and can contribute to the suppression of innate immunity, RNA silencing, post-transcriptional gene silencing, and transcriptional gene silencing [41,42,43]. This protein is the most diverse among the *Geminiviridae* family and undergoes various post-translational modifications, including N-myristoylation, S-acylation, and phosphorylation [44]. This remarkable diversity likely contributes to the functional variability observed among C4 proteins from different geminivirus species. N-myristoylation and palmitoylation are required for preferential binding to the plasma membrane [45,46]. Post-translational modifications have not yet been associated with C4 movement to the chloroplasts, but the movement is aided by the β-subunit of the COAT PROTEIN COMPLEX I (COPI) [47]. Localization of the C4 protein in chloroplasts suppresses salicylic acid production, thereby sabotaging the SA defense pathway [48].

DNA methylation requires a methyl group. DNA methyltransferases (DNMTs) donate the methyl group from S-adenosylmethionine (SAM). SAM is produced by a synthetic reaction from methionine and ATP, catalyzed by SAM synthetase (SAMS). Several studies of the C4 protein of Bhendi yellow vein mosaic virus, Tomato yellow leaf curl China virus (TYLCCNV), and Cotton leaf curl Multan virus (CLCuMuV) have shown that C4 reduced methylation by interacting with the SAMS protein [43,49,50,51]. Ismayil et al. [43] mapped out that the C4 protein binds to the N-terminus of SAMS, where Arginine 13 (R-13) is an essential residue for this interaction. In the viruses examined to date, C4 suppresses SAMS activity, thereby impairing the host’s methylation capacity.

Interestingly, the C4 protein can interact with both plant and vsiRNAs, which interferes with post-transcriptional gene silencing (PTGS) and transcriptional gene silencing (TGS) [52,53,54]. Cabbage leaf curl virus (CaLCuV) and African cassava mosaic virus (ACMV) generate 21- to 24-nt vsiRNAs using Dicer-like (DCL) proteins. HEN1 methylation stabilizes these vsiRNAs [55]. The C4 protein inhibits HEN1’s access to vsiRNAs by binding and sequestering unmethylated vsiRNAs, thereby reducing the pool of AGO-bound vsiRNAs. In contrast, the Tomato leaf curl New Delhi virus C4 protein does not interact with any vsiRNAs or their duplexes; instead, it interacts with AGO4, impairing its function [56]. This highlights the distinct mechanisms by which begomoviral species perturb upstream DNA methylation. These observations suggest that C4 proteins suppress methylation-based defenses in a mechanistically conserved way, yet may use distinct molecular interactions across geminivirus species.

Domains Rearranged Methyltransferase 2 (DRM2) is an N-methyltransferase that catalyzes the de novo methylation of all 3 islands. It comprises an N-terminal region with multiple ubiquitin-associated (UBA) motifs and a C-terminal methyltransferase domain. Unlike UBA domains in other proteins, whose primary function is protein degradation, DRM2’s UBA domains have evolved a specialized role in RdDM by facilitating the targeting and recruitment of DNA methylation machinery to specific genomic locations [57,58]. Both domains are needed for the enzyme’s catalytic activity. Furthermore, enzyme function depends on homodimerization via the C-terminal domain [58]. The NbDRM2 protein interacts with the Tomato leaf curl Yunnan virus C4 protein via serine 14 (S-14) [59]. Formation of a NbDRM2/C4 complex prevents DRM2 from binding DNA and transferring methyl groups, thereby reducing overall methylation of viral DNA. Beet curly top virus and CaLCuV-infected plants showed increased susceptibility when specific RdDM genes (e.g., *drm2*, *ago4*, *dcl*, and *rdr* enzymes) were silenced [28]. The mRNA transcript level of NbDRM2 was not affected by Tomato yellow leaf curl Sardinia virus, Tomato golden mosaic virus, and a mild strain of Tomato yellow leaf curl virus; however, ACMV showed reduced levels [38]. Interestingly, C4 may cooperate with C1 to suppress host DNA methylation by downregulating MET1 [38].

Overall, these findings demonstrate that C4 proteins suppress methylation-based antiviral defenses at multiple levels, including methyl donor biosynthesis, small RNA-mediated targeting, and the catalytic activity of DNA methyltransferases (Table 2). The diversity of these mechanisms reflects both the multifunctional nature of C4 proteins and their extensive evolutionary divergence across the *Geminiviridae*.

### 3.3. Transcription Activator Protein (C2)

The A/C2 ORF encode the geminivirus Transcription activator protein (TrAP), and its promoter is located within the C1 ORF. The C2 protein transactivates coat and movement proteins (late genes). It also suppresses plant defense and acts as a virus pathogenicity factor [23]. The C2 protein is about 15 kDa and consists of 129–145 amino acids. The protein can exist in either a phosphorylated form in the nucleus or a non-phosphorylated form in the cytoplasm [60]. It is a modular protein with a basic nuclear localisation domain (N-terminal) and an acidic C-terminal domain that mediates transcriptional activation. A central zinc finger-like motif separates the two domains and suppresses programmed cell death. Curtoviruses and begomoviruses share little intergenic sequence similarity, except for conserved central cysteine-histidine residues [61]. Unlike begomoviruses, the curtoviruses lack the acidic C-terminal domain. Therefore, late-gene activation depends on cell-cycle reactivation, which induces expression of host DNA replication and G2/M transition genes [62]. Furthermore, C2 has a low affinity and no specificity for plant DNA [60,63]. Consequently, C2 exerts many of its functions through interactions with host regulatory proteins that influence defense, transcription, and chromatin dynamics.

**Figure 1:**
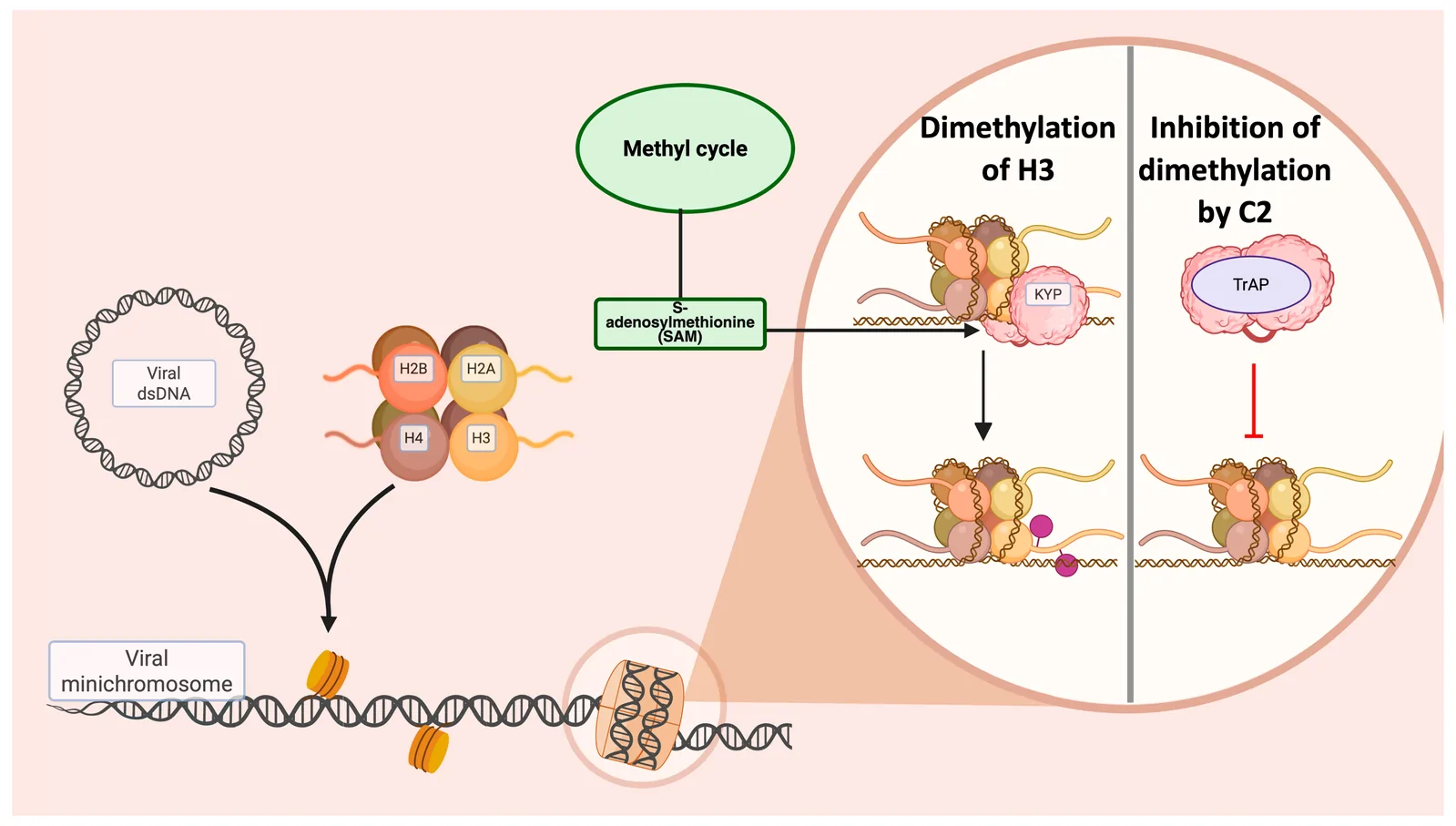
Geminivirus C2-mediated inhibition of viral minichromosome dimethylation. Host histones (H2A, H2B, H3, and H4) wrap viral double-stranded DNA (dsDNA) to form viral minichromosomes, which serve as templates for viral replication and transcription. Under epigenetic defense, the histone methyltransferase KRYPTONITE (KYP) represses viral chromatin activity through dimethylation, using S-adenosylmethionine (SAM) as a methyl donor. The geminivirus-encoded transcriptional activator protein (C2), represented by a purple circle, counters this by directly binding and inhibiting KYP activity, thereby preventing the induction of host-mediated TGS, which would otherwise promote continuous viral gene transcription and replication.

Like C4-mediated suppression of SAM activity, geminiviral C2 proteins can reduce methylation capacity by disrupting enzymes required to maintain the methyl cycle. Adenosine kinase (ADK) is a conserved enzyme that phosphorylates adenosine to adenosine monophosphate (AMP), maintaining cellular adenylate pools and supporting the SAM-dependent methyl cycle essential for DNA and histone methylation. In plants, ADK is required for normal methylation homeostasis and cytokinin metabolism, and loss of ADK function misregulates methylation-dependent pathways [64,65]. Arabidopsis mutant plants defective in ADK-encoding genes were hypersensitive to geminivirus infection [28]. Buchmann and colleagues [66] showed that AL2 and L2, positional homologs of AC2 and C2 in bipartite and monopartite geminiviruses, directly interact with ADK. The interaction between AL2/L2 and ADK disrupts methyl-cycle homeostasis, thereby indirectly reducing methylation capacity and the efficiency of DNA methyltransferase-mediated reactions. This led to reduced global methylation of non-CG islands and increased viral transcription. Both ADK and SAMDC are vital for maintaining methyl-cycle homeostasis. ADK removes adenosine by phosphorylating it back to AMP using adenosine triphosphate (ATP), thereby promoting continued SAM production. SAMDC then uses SAM to synthesize polyamines. SAMDC is constitutively produced in plants and undergoes rapid ubiquitin-dependent degradation by the 26S proteasome [67]. SAMDC decarboxylates SAM to form dcSAM. The SAM/dcSAM ratio influences host transmethylation; high dcSAM levels may inhibit DNMTs, as dcSAM acts as a competitive inhibitor of DNMTs [68]. The BCTV C2 protein can bind the PEST (proline-, glutamic acid-, serine-, and threonine-rich region; amino acids 226 and 292) of the SAMDC1 protein, a region associated with rapid turnover [65]. The interaction between BCTV C2 and SAMDC1 attenuates proteasome-mediated SAMDC1 degradation, increasing SAMDC1 accumulation in infected plants. This may increase dcSAM concentration in infected plants, thereby altering the SAM/dcSAM ratio in infected cells. Furthermore, by manipulating the SAM/dcSAM ratio, geminivirus C2 disrupts the RdDM reinforcing loop by decreasing cytosine methylation in the 3 sequences and by reducing siRNA accumulation [65,69]. Geminivirus C2 proteins suppress methylation-based antiviral defenses mainly indirectly, by blocking upstream metabolic and chromatin pathways that RdDM depends on, rather than by directly interacting with RdDM proteins. In addition to this metabolic interference, C2 also impacts chromatin remodelling by directly binding and inhibiting the H3K9me2 histone methyltransferase, Su(var)3-9 homolog 4/Kryptonite (SUVH4/KYP). KYP deposits repressive H3K9me2 marks on the viral minichromosome. Therefore, C2-mediated inhibition of KYP activity interferes with KYP-dependent CHH DNA methylation (Figure 1) [30].

Overall, these findings demonstrate that C2 suppresses methylation-based antiviral defenses through both metabolic and chromatin-associated mechanisms (Table 2). By disrupting methyl-cycle homeostasis through ADK and SAMDC and simultaneously inhibiting KYP-dependent histone methylation, C2 reduces RdDM efficiency. It thus promotes a chromatin environment more permissive for viral gene expression.

### 3.4. Movement Protein (V2)

In monopartite begomoviruses, V2 functions as a movement-associated protein, whereas its positional homolog AV2 in certain bipartite viruses is commonly called the pre-coat protein. Unlike the C1 protein, V2 is absent in some geminiviruses, such as native old-world begomoviruses. Interestingly, the movement protein of bipartite geminivirus, encoded by the BC1 ORF, has not been identified as a suppressor of plant defense. Curtovirus and begomovirus V2 proteins are thought to have evolved convergently because they show low sequence similarity yet share conserved functional domains, are functionally homologous, and target identical pathways [70]. Sequence alignment showed that begomoviruses and curtoviruses contain a protein kinase CK2/protein kinase C phosphorylation motif and two hydrophobic N-terminal domains, followed by a long hydrophilic C-terminal domain. However, curtoviruses lack the CxC motif that is conserved in the begomovirus genus [70]. The V2 protein is multifunctional; it is required for viral movement, systemic movement, and suppression of PTGS and TGS [71,72,73,74,75,76,77,78]. It can also modulate hypersensitive response-like defenses by interacting with the papain-like cysteine protease CYP1 and Catalase 2 [79,80].

The V2 protein localizes to the cytoplasm, where it impedes RNA silencing by interacting with the Suppressor of Gene Silencing (SGS3) protein, and promotes accumulation of the geminivirus C4 protein by associating with the endoplasmic reticulum and the nucleus, sites of TGS suppression [81,82,83]. Beyond suppressing PTGS, V2 also directly interferes with RdDM components responsible for transcriptional gene silencing. The ClCuMuV V2 protein enhances viral infection and hinders RdDM by interacting with AGO4. The direct interaction between ClCuMuV V2 and NbAGO4 interferes with AGO4-mediated antiviral silencing and contributes to RdDM suppression [84]. Additionally, TYLCV V2 suppressed TGS by binding to AGO4 in the nuclear Cajal body, where V2 association with AGO4 did not hamper vsiRNA production [84]. Interestingly, an L76S mutation disrupted the interaction between ClCuMuV V2 and NbAGO4; the TYLCV V2 mutant did not abolish this interaction with NbAGO4 or SlAGO4. Rather, the L76S mutation disrupted V2 self-interaction in the Cajal body [84,85]. Histone deacetylase 6 (HDA6) contributes to TGS maintenance by physically interacting with MET1 to cooperatively mediate gene silencing. During TYLCV infection, the V2 protein interacts with HDA6 but not with NbMET1. Interestingly, TYLCV V2 does not appear to directly inactivate HDA6. Instead, V2 interferes with the interaction between HDA6 and MET1, thereby impairing HDA6-mediated recruitment of MET1 to viral chromatin. This disruption reduces viral DNA methylation and enhances host susceptibility to infection [82]. Collectively, these studies demonstrate that V2 suppresses methylation-based antiviral defenses through multiple complementary mechanisms. By targeting AGO4-dependent RdDM and disrupting HDA6-mediated recruitment of methylation machinery, V2 interferes with both the establishment and maintenance of transcriptional gene silencing. These findings further illustrate how geminiviruses have evolved diverse suppressors that target distinct nodes of the host epigenetic defense network (Table 1).

## 4. Can Geminivirus Satellites Disturb RdDM?

**Figure 2:**
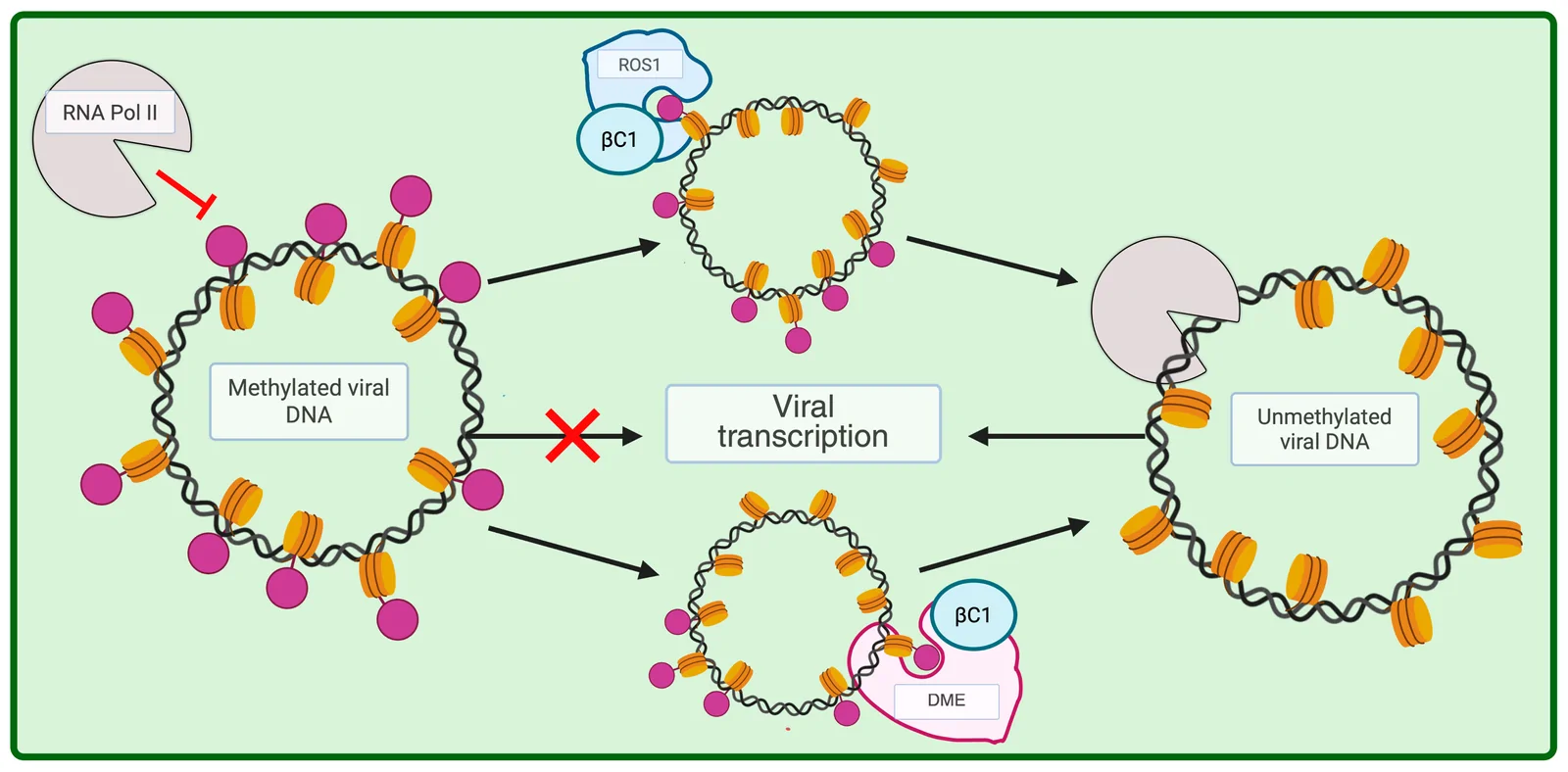
Geminivirus βC1 interacts with ROS1 and DME to promote demethylation. The geminivirus βC1 (shown in green circles) protein exploits the demethylation pathway by interacting with the host demethylases Repressor of silencing 1 (ROS1) and Demeter (DME). Methylation of viral minichromosomes inhibits RNA polymerase II (RNA Pol II) binding to the viral chromatin. Direct interaction of βC1 with these demethylase enzymes enhances the host’s active demethylation pathways, removing repressive epigenetic marks from viral minichromosomes. This restores minichromosomes to an open state, enabling RNA Pol II to bind and facilitate viral transcription.

Some geminiviruses have accessories, subviral ssDNA elements called satellites. Satellites rely on the helper virus for their replication, encapsidation, and movement. Satellites include α-, β-, δ-, and Ɣ-satellites, as well as Sequence Enhancing Geminivirus Symptoms 2 (SEGS-2), which occur exclusively in Cassava plants [86,87,88,89,90]. Most geminivirus satellites depend on the helper virus for replication, except for the α-satellites, which can replicate autonomously. α- and β-satellites share an adenine-rich sequence absent from other geminiviral satellites. Geminivirus α-satellites encode a replication protein, whereas β-satellites encode a pathogenicity factor and, on the C1 ORF, a suppressor of viral defense protein. However, TYLCCNV was recently reported to contain an additional ORF on the virion-sense strand that encodes a symptom-determinant protein (βV1) [91]. TYLCCNV βC1 reduces global methylation in *N. benthamiana* plants and reverses TGS in *N. benthamiana* and Arabidopsis plants. βC1 can interact with and partially inhibit S-adenosylhomocysteine hydrolase (SAHH) [33]. SAHH is a regulatory enzyme that facilitates the catabolism of S-adenosylhomocysteine (SAH) into adenosine and homocysteine, a by-product of SAM-dependent methylation. Inhibiting SAHH may increase SAH levels, which competitively bind methyltransferases and prevent SAM from binding [92]. Like TrAP-mediated disruption of ADK and SAMDC, SAHH inhibition perturbs methyl-cycle homeostasis and reduces the efficiency of methyltransferase-dependent reactions. Interestingly, βC1 is currently the best-characterized geminivirus-encoded suppressor reported to exploit host DNA demethylation pathways. βC1 can interact with DNA glycosylases such as REPRESSOR OF SILENCING 1-like (ROS1L) and DEMETER (DME) to promote virulence and the hypomethylation of viral DNA. βC1’s direct interaction with DNA glycosylases enhances their enzymatic activity, thereby counteracting TGS [25]. Interestingly, phosphorylation of βC1 inactivates its ability to reverse TGS and attenuates viral symptoms (Figure 2) [93].

**Table 2:** Role of geminivirus protein in the suppression of DNA methylation.

Viral Protein	Host Target	Effects of DNA Methylation/Epigenetic Regulation	Reference
C1	UBC2	Increases the deposition of H2B-ub, leading to the promotion of trimethylation of histone 3 at lysine 4	[37]
	HUB1		
	MET1	Reduces transcription of DNA methyltransferase	[38]
	CMT3		
	VIM5	Promotes the ubiquitination of MET1 and CMT3 by increasing expression of VIM5	[39]
C4	SAMS	Inhibits SAMS activity	[43,50,51,52]
	DRM2	Prevents the transfer of methyl groups to DNA by inhibiting DRM2 binding to DNA	[59]
	AGO4	Inhibits AGO4 activity by blocking vsiRNAs binding to AGO4	[56]
V2			[84]
	HDA6	Prevents the recruitment of MET1 by HDA6	[92]
C2	SAMDC1	Attenuates the degradation of SAMDC1	[65]
	ADK	Suppresses ADK activity	[66]
	KYP	Antagonizes KYP-mediated histone methylation by inhibiting KYP activity	[30]
βC1	SAHH	Reduces methyltransferase efficiency by increasing SAH accumulation	[33,92]
	ROS1L	Promotes DNA demethylation by enhancing the enzyme activity of ROS1L and DME	[93]

## 5. Can DNA Methylation Disturb Geminiviral Infection?

**Figure 3:**
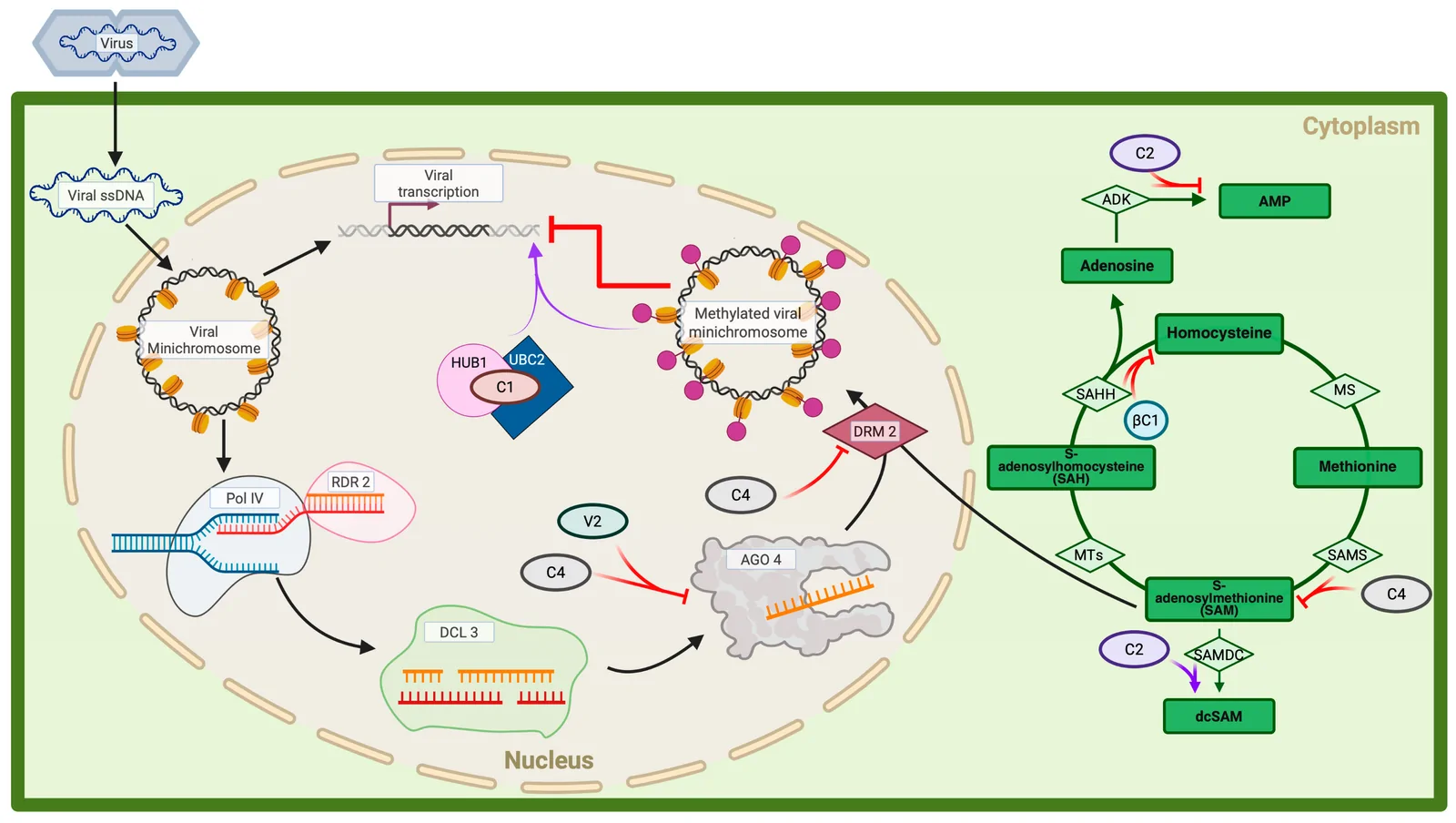
DNA methylation mediates transcriptional gene silencing (TGS) of geminiviral minichromosomes, whereas geminivirus suppressor proteins attenuate TGS by associating with DNA methylation and methyl cycle components at multiple nodes. Black arrows denote the DNA methylation pathway, green arrows illustrate the methyl cycle, red T-arrows represent inhibition by viral suppressor proteins, and purple arrows denote pathway diversion by viral suppressor proteins to impair DNA methylation. Pol IV, RNA polymerase IV; RDR2, RNA-dependent RNA Polymerase 2; DCL 3, Dicer-like 3; AGO 4, Argonaute 4; DRM 2, Domains rearranged methyltransferase 2; HUB, histone monoubiquitination 1; UBC2, Ubiquitin-conjugating enzyme 2; ADK, Adenosine kinase; AMP, Adenosine monophosphate; SAHH, S-adenosylhomocysteine hydrolase; MS, Methionine synthase; SAMS, S-adenosylmethionine synthase; dcSAM, Decarboxylated S-adenosylmethionine; SAMDC, S-adenosylmethionine decarboxylase; MTs, Methyltransferases; C1, Replication-associated protein (brown circle); C2, Transcription Activator protein (purple circle); V2, Movement protein (dark green circle); β-C1, Betasatellite-encoded pathogenicity factor. The figure illustrates how geminiviral suppressors target multiple interconnected components of the host methylation machinery, highlighting the coordinated nature of viral counter-defense strategies against RdDM-mediated antiviral immunity.

Geminiviruses use a range of mechanisms to exploit host pathways, subvert defenses, and promote replication. However, plant defense strategies remain effective. Brough et al. [94] found that increased m5C methylation of TGMV DNA reduced viral accumulation. The decreased viral accumulation was associated with reduced viral replication efficiency. Furthermore, viral progeny DNA did not retain in vitro-introduced methylation marks, showing that TGS of viral DNA requires continued maintenance by host-directed methylation machinery. Similarly, Ermark and co-workers [95] reported that in vitro homomethylated viral DNA negatively affected African cassava mosaic virus (ACMV) DNA replication efficiency. A co-infection study between Tomato yellow leaf curl Sardiana virus (TYLCSV) and potato spindle tuber viroid (PSTVd) demonstrated that the PSTVd antagonized TYLCSV infection and viral accumulation by inducing hypermethylation of TYLCSV DNA and the upregulation of genes involved in cellular methylation metabolism (ADK, SAHH, and SAMDC1), *de novo* methylation (HEN1, DRM2, AGO4, and NRPE1), histone methylation (IBM1 and KYP), maintenance methyltransferases (MET1, DDM1, and CMT3), and demethylation enzyme ROS1 [96]. BCTV *L2*- null mutants recovered from infection had a hypermethylated intergenic region, whereas *ago4*-deficient plants did not recover [26]. Recovery from geminiviral infection is associated with a compact chromatin structure. *A. thaliana nrpdb-11* mutant plants, devoid of the large catalytic subunit of Pol V, infected with BCTV *L2^−^* null mutants did not recover and had a lower level of compact structures compared to wild-type recovered plants, which had approximately 90% of compact viral chromatin [97]. *Capsicum annuum* L. var. Sonora Anaheim recovered from Pepper golden mosaic virus showed milder symptoms or none, linked to reduced viral titer and hypomethylation of the geminivirus IR [98]. Furthermore, minichromosome structure and epigenetic density analysis revealed that minichromosomes isolated from recovered *Capsicum annuum* L. var. Sonora Anaheim tissue exhibited a condensed structure associated with higher levels of the chromatin-repressive marker (H3K9me2) and higher DNA methylation (Figure 3). Conversely, minichromosomes from symptomatic tissue showed a relaxed conformation associated with higher levels of the chromatin-repressive marker (H3K9me32) and lower DNA methylation [99]. Similarly, Mulaudzi et al. [8] found that ToCSV-tolerant plants had higher methylation levels than susceptible plants. Both ToCSV-infected cultivars showed upregulation of DNA methylation (*AGO4*, *AGO6*, and *RDM1*), DNA demethylase (*DML2*), and histone modification (*KYP/SUVH4*) genes; however, susceptible plants showed downregulation of *MET1*. *MET1* repression was associated with suppressed CHG maintenance and lower DNA methylation levels [8].

Taken together, these findings indicate that geminivirus-encoded suppressor proteins such as C1, C2, C4, V2, and βC1 suppress methylation-based antiviral defenses by both inhibiting the methyl cycle and activating host DNA demethylation pathways. Unlike the viral suppressor proteins discussed above, βC1 not only reduces methylation capacity but may also actively promote the removal of existing methylation marks from viral DNA, thereby enhancing viral gene expression and infection.

## 6. Conclusions

RNA-directed DNA methylation constitutes an important component of the host defense network that restricts geminivirus infection through transcriptional gene silencing and chromatin-based repression of viral genomes. This review highlights that DNA methylation is a key regulator of geminiviral infection and is therefore actively targeted by geminiviral proteins. RdDM negatively affects viral gene expression and replication, thereby reducing viral DNA accumulation. Furthermore, host recovery has been associated with TGS and compact viral chromatin configuration. Although the complete geminivirus–host interactions focused on epigenetic regulation remain incompletely understood, many aspects of geminivirus–host interactions involving epigenetic regulation remain fully elucidated. Evidence indicates that geminiviral proteins act in complementary and auxiliary roles to subvert DNA methylation. Counterdefense of RdDM includes disrupting the methyl cycle by decreasing SAM availability, sequestering vsiRNAs from AGO4-mediated processing, hindering protein function through direct interactions, and transcriptionally repressing DNMTs. Although these suppressors act through distinct mechanisms, they collectively target multiple components of the host methylation machinery, highlighting the importance of DNA methylation as an antiviral defense against geminivirus infection. βC1 protein acts as an accessory modulator of RdDM suppression by increasing SAH accumulation and exploiting the DNA demethylase pathway to promote hypomethylation of viral DNA. The effects of DNA methylation on C1 DNA-binding affinity and endonuclease activity remain unclear. Furthermore, the relative contribution of individual suppressors to methylation suppression during natural infection remains unclear and warrants further investigation. A clearer understanding of how DNA methylation inhibits viral replication will provide insights into host–geminivirus interactions and host antiviral defense, and may support the development of disease control strategies.

## Figures and Tables

**Table 1:** Nomenclature and abbreviations of geminivirus-encoded proteins.

Monopartite	Bipartite Counterpart	Protein
C1	AC1/AL1	Replication-associated protein (Rep)
C2	AC2/AL2	Transcriptional Activator protein (TrAP)
C4	AC4/AL4	C4 protein, pathogenicity and defense suppressor protein
V2	AV2	Movement protein; bipartite positional homolog of the pre-coat protein.

## Data Availability

All source data generated in this study are available from the corresponding author upon request.
